# Disitamab Vedotin plus anti-PD-1 antibody show good efficacy in refractory primary urethral cancer with low HER2 expression: a case report

**DOI:** 10.3389/fimmu.2023.1254812

**Published:** 2023-10-12

**Authors:** Yue Zheng, Yin-Yin Xue, Ya-Qin Zhao, Ye Chen, Zhi-Ping Li

**Affiliations:** ^1^ Department of Biotherapy, State Key Laboratory of Biotherapy, West China Hospital, West China Medical School, Sichuan University, Chengdu, Sichuan, China; ^2^ Department of Radiation Oncology, West China Hospital, Sichuan University, Chengdu, Sichuan, China; ^3^ Department of Medical Oncology, Cancer Center, West China Hospital, Sichuan University, Chengdu, Sichuan, China; ^4^ Lung Cancer Center, West China Hospital, Sichuan University, Chengdu, Sichuan, China; ^5^ Division of Abdominal Tumor Multimodality Treatment, West China Hospital, Sichuan University, Chengdu, Sichuan, China

**Keywords:** advanced primary urethral carcinoma, antibody drug conjugate, HER2, immunotherapy, PD-1 inhibitor

## Abstract

Primary urethral carcinoma (PUC) has a low incidence, but with high aggressiveness. Most of the patients are found in late stage, with poor prognosis. At present, chemotherapy is still the main treatment for metastatic PUC, but it has limited effect. Here, we report a case of metastatic PUC with low HER2 expression that developed disease progression after multiline therapy including chemotherapy, programmed death-1 (PD-1) inhibitors and multi-targeted receptor tyrosine kinase (RTK) inhibitor. After receiving Disitamab Vedotin(a novel antibody drug conjugate, ADC) and toripalimab (a PD-1 inhibitor), the patient achieved persistent PR, and the PFS exceeded 12 months up to now. Our report indicates that, despite the patient of metastatic PUC has low expression of HER2, it is still possible to benefit from Disitamab Vedotin combined with PD-1 inhibitor, which may reverse the drug resistance of PD-1 inhibitor and chemotherapy to a certain extent. But larger sample studies are needed to determine the efficacy of this treatment strategy and its impact on survival.

## Introduction

Primary urethral carcinoma (PUC) is a rare malignancy, accounting for less than 1% of all malignancies (1). Primary female urethral cancer is even rarer, accounting for less than 0.02% of all female malignancies (2, 3). According to the European Association of Urological Surgery (EAU) Guidelines for Primary urethral cancer, a PUC is defined as first carcinoma in the urinary tract that originates from the urethra (4). The main pathological types include squamous cell carcinoma, transitional cell carcinoma, adenocarcinoma and other rare pathological types (3, 5, 6). Metastatic PUC has a poor prognosis. The reported 5-year survival rate for female primary urethral cancer ranged from 32% to 54% (7–9). Due to the low incidence of PUCs, no statistics on survival of metastatic PUCs are available. Metastatic PUC lacks optimal treatment strategies based on current study data, chemotherapy is the main treatment, and multi-mode therapy is recommended (10, 11). The efficacy of various therapeutic strategies, including immunotherapy and targeted therapy, has only been reported in a few individual cases. New effective therapeutic drugs and strategies need to be further explored. Disitamab Vedotin is a novel antibody drug conjugate (ADC) independently developed in China. It has been approved for use in patients with locally advanced or metastatic uroepithelial carcinoma who have received platinum-containing chemotherapy in the past and have overexpression of HER2 (HER2 immunohistochemical results are 2+ or 3+) (12).

In this article, we report the efficacy of Disitamab Vedotin in combination with toripalimab (anti-PD-1 antibody) in metastatic primary female urethral cancer with disease progression after multiline therapy. The patient had low expression of HER2 and received chemotherapy, anti-PD-1 monoclonal antibody and targeted therapy, but disease progression occurred after these treatments. The treatment regimen of Disitamab Vedotin combined with toripalimab enabled the patient to obtain persistent PR. So far, the patient has not experienced disease progression, and the PFS has exceeded 12 months.

## Case report

**Figure 1:**
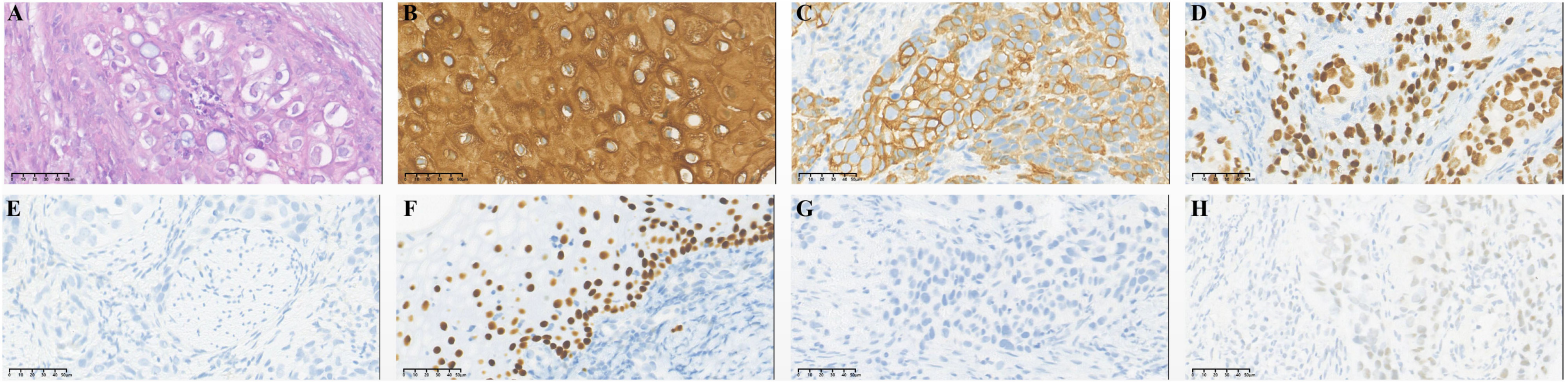
Immunohistochemical staining of primary tumor: urethral carcinoma. **(A)** Hematoxylin-eosin staining of urethral carcinoma. Tumor cells were positive for CK5/6 **(B)**, CK7 **(C)** and GATA-3 **(D)** and negative for CK20 **(E)**, P63 **(F)**, Uroplakint-2 **(G)** and Uroplakint-3 **(H)**.

A 54-year-old female was admitted with intermittent urethral bleeding and urinary tract ultrasonography revealed urethral tumor. Puncture biopsy for urethral tumor was performed. And pathology examination revealed malignant tumor of urethra (poorly differentiated carcinoma) supported by immunohistochemical staining as follows: CK7 (+), GATA-3 (+), CK5/6 (+), P63 (-), CK20 (-), DX-2 (-), ER (-), PR (-), Uroplakint2 (-), Uroplakin-3 (-), HPV (-) (**Figure 1**). Due to the low differentiation of tumors, it is difficult to further determine the direction of differentiation. The patient underwent radical urethrotomy (R1 resection). Intraoperative findings: new organisms were found in the outer orifice of the urethra, the anterior wall of the vagina was stiff, the tumor invaded the bulbocavernosum muscle, the posterior urethra and part of the anterior wall of the vagina, and no tumors were found in the walls of the bladder and trigone. After surgery, the patient received 7 cycles of adjuvant chemotherapy with GP regimen (gemcitabine 1000mg/m2 day1,8 q3w and cisplatin 75mg/m2 day1 q3w) and VMAT technology radiotherapy (60Gy/30f) for the urethral stump tumor bed.

**Figure 2:**
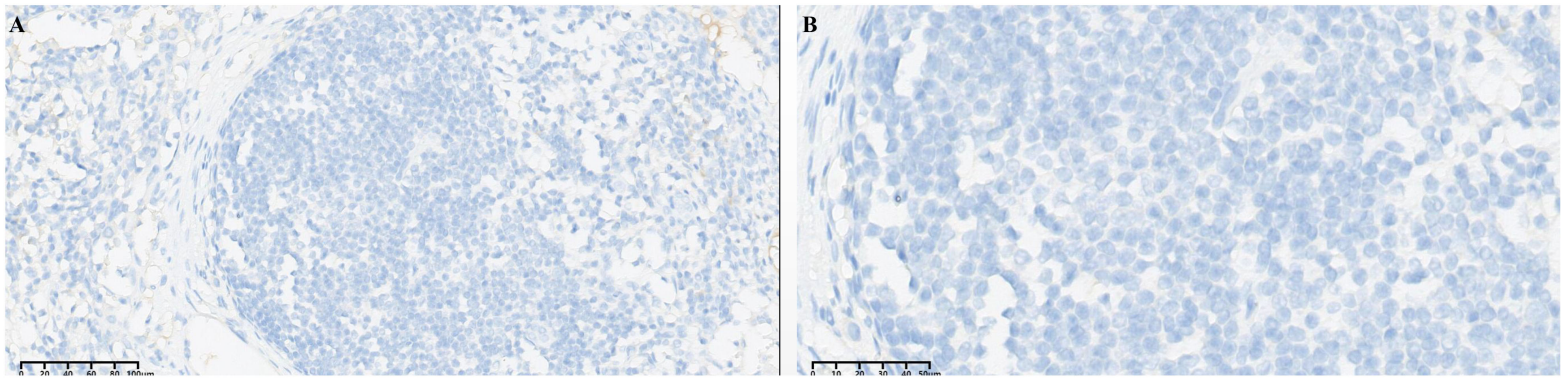
Immunohistochemical staining 20X **(A)**, 40X **(B)** depicted HER2 (1+) in tumor cells.

Bilateral inguinal lymph node metastases were observed 5 months after completion of adjuvant treatment. The patient underwent bilateral inguinal lymph node dissection. A total of five lymph nodes showed cancer metastasis, and the immunohistochemical results were as follows: MLH1 (+), MSH2 (+), MSH6 (+), PMS2 (+), HER2 (1+, referring to the evaluation criteria of breast cancer HER2), Ki-67 (+, 80%) (**Figure 2**). The patient received radiotherapy (60.2Gy/28f) in the inguinal region. The pan-cancer 1021 gene detection of solid tumors showed 7 somatic mutations and 0 germline mutation. The results of immune checkpoint inhibitor molecular markers showed BRCA2 mutations and CHEK2 mutations, which may be related to the benefit of PD/PD-L1 inhibitors. Tumor mutation burden was 2.88 Muts/Mb (TMB-L). PD-L1 expression in the tumor cell and tumor vasculature was TPS<1%, CPS 2-3. Immunotherapy with tislelizumab (200mg ivgtt q3w) was used as second-line treatment. After 3 cycles and 7 cycles, the efficacy was evaluated as stable disease (SD). After 12 cycles, enhanced CT showed new multiple metastases in bilateral lungs, neck, abdominal and pelvic lymph nodes. The efficacy was evaluated as progression disease (PD). The patient then volunteered to participate in a phase 1 clinical trial for ICP-033 (1mg po qd), a novel multitargeted receptor tyrosine kinase (RTK) inhibitor. Three months after the patient took ICP-033 orally, all lesions increased or enlarged. The comprehensive efficacy evaluation was PD. And the patient had significant pelvic pain. The patient had a BRCA2 mutation but had experienced a grade IV myelosuppression during previous chemotherapy, so PARP (poly ADP-ribose polymerase) inhibitors that could cause severe myelosuppression were excluded during treatment selection.

**Figure 3:**
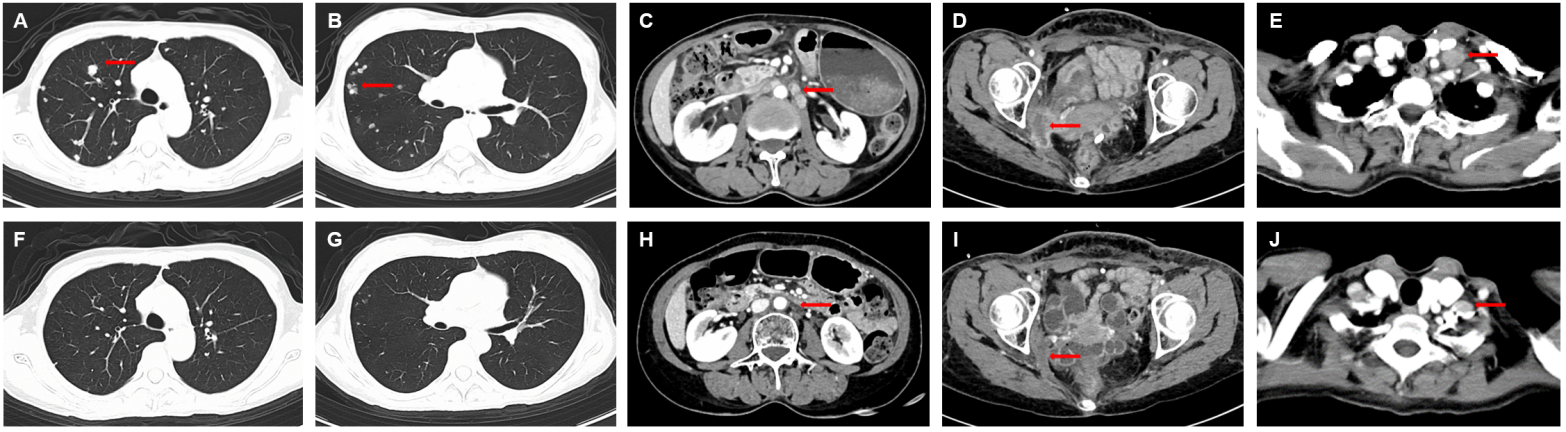
Computed tomography scan before and after Disitamab vedotin combined with toripalimab treatment. **(A-E)** Lung, pelvic lymph node and left supraclavicular lymph nodemetastases before treatment with Disitamab vedotin in combination with toripalimab. **(F-J)** All lesions were significantly reduced after 18 cycles of combined treatment. The red arrows indicate the lesions.

**Figure 4:**
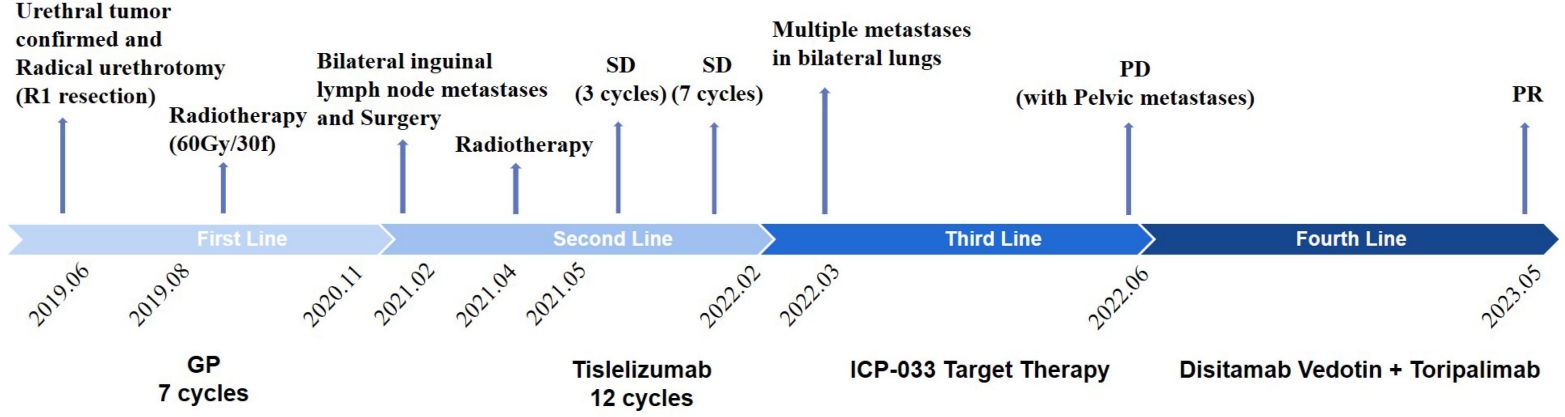
Timeline scheme of major clinical event of the patient since diagnosis.

Disitamab Vedotin combined with toripalimab (toripalimab 135mg, ivgtt+ Disitamab Vedotin 90mg, ivgtt, day 1, every 2 weeks) was used as fourth line treatment. Pelvic pain symptoms were significantly relieved after the first cycle of treatment. After 3 cycles of combined treatment, bilateral lung and neck, abdominal and pelvic lymph nodes were significantly reduced, and the comprehensive efficacy was evaluated as partial response (PR). To date, the patient has received Disitamab Vedotin combined with toripalimab 19 cycles and had an ongoing response for more than 12 months (**Figure 3**). Most importantly, minor adverse events, including grade II leukopenia and grade I neurotoxicity, were observed during the treatment. No other treatment-related adverse events such as infusion reaction, cardiotoxicity, pulmonary toxicity and hepatic toxicity occurred during treatment. These symptoms were improved after symptomatic treatment. The patient’s treatment process is shown in **Figure 4**.

## Discussion

As a rare tumor type, there is no standard of optimal treatment for patients with advanced primary urethral carcinoma. Currently, based on limited evidence and different focal locations or pathological features, multi-mode therapy (including high-quality surgery, chemotherapy, radiotherapy and the above combined therapy) is the main treatment strategy for primary urethral cancer, especially for patients with advance stage (4, 13). Several studies have reported that different pathologic types of primary urothelial carcinoma have different sensitivities to different therapies. Son et al. retrospectively analyzed the survival outcomes of 2614 patients with urethral cancer. It has been found that for patients with advanced PUC, non-adenocarcinoma or transitional cell carcinoma may benefit from surgery combined with radiation therapy as a treatment strategy (14). Peyton et al. reported that multi-mode therapy did not significantly improve the survival rate of primary female urethral cancer, but this may be related to the small sample size of this study (10).

Currently, immunotherapy, especially programmed death receptor-1 (PD-1)/programmed death ligand (PD-L1) blockers, have shown encouraging results in the treatment of a variety of advanced solid tumors. PD-1/PD-L1 blockers have been approved for second-line treatment of metastatic urothelial carcinoma after first-line platinum-based therapy has failed. Results from KEYNOTE-045 showed that Pembrolizumab achieved a longer median survival time and a higher response rate in advanced patients who progressed after platinum-based therapy compared to chemotherapy. For patients with PD-L1(+) who are not suitable for cisplatin, pembrolizumab or atezolizumab can be selected as first-line therapy. However, the evidence is for metastatic urothelial carcinoma, including bladder, upper urinary tract, and urethral cancers. Immunotherapy for PUCs has only been reported in a small number of cases. However, for patients with advanced PUC after advanced multiline therapy, immunotherapy be an alternative strategy to try.

Antibody drug conjugate (ADC) is a class of targeted biological drugs composed of highly targeted monoclonal antibody, junction head and cytotoxic drugs. Using monoclonal antibody as a carrier, small molecule cytotoxic drugs can be efficiently transported to the target tumor cells to play an anti-tumor role (15). ADC has both targeting and drug toxicity. Currently, a number of ADC drugs have been approved and marketed for antitumor therapy, among which Enfortumab vedotin (EV), Sacituzumab govitecan-hziy (SG) and Disitamab Vedotin are used for uroepithelial carcinoma. Disitamab Vedotin targets HER-2 and consists of Hertuzumab (a novel anti-HER2 monoclonal antibody) coupled to monomethyl auristatin E (MMAE, a cytotoxic drug) via a lysable link (16, 17). Results from the Phase II RC48-C009 study showed an objective response rate (ORR) of 50.0% treated with Disitamab Vedotin in locally advanced or metastatic uroepithelial carcinoma with HER-2 overexpression that had failed previous chemotherapy (12). Results of a Phase I clinical study showed that antitumor response of Disitamab Vedotin in patients with gastric cancer with low HER2 expression (IHC2+/FISH-) was similar to that of HER2 IHC2+/FISH + and IHC3+. Significant tumor shrinkage was achieved in 72.7%, 60.0% and 52.6% of patients, respectively (18). Results from a single-arm Phase II clinical study (NCT04073602) showed an objective response rate (ORR) of 26.3% and a disease control rate (DCR) of 94.7% in uroepithelial carcinoma patients who had previously received at least one systemic therapy with low HER2 expression (IHC 0 or 1+).

A preclinical study found that in a hHER-2 transgenic mouse model, Disitamab Vedotin combined with PD-1/PD-L1 immune checkpoint inhibitors could significantly enhance T-cell-related anti-tumor immunity, enhance tumor suppression, and also contribute to the formation of immune memory (19). At present, some clinical studies have also been carried out on the combination therapy of Disitamab Vedotin and immunotherapy. Li et al. presented clinical study data of NCT04264936 at the 2022 ASCO annual meeting. In patients with locally advanced or metastatic urothelial carcinoma with HER2 (+/-), Disitamab Vedotin in combination with toripalimab had an ORR of 75% in confirmed investigator assessments at study cut-off. The ORR was 66.7% for HER2 (1+), and 50% for HER2 (0) respectively (20). A retrospective study showed that patients with locally advanced or metastatic uroepithelial carcinoma may benefit from the use of Disitamab Vedotin combination immunotherapy (21).

Based on these evidences, the strategy of combination of Disitamab Vedotin and toripalimab was selected for this patient. The treatment results showed that the lung and neck, abdominal and pelvic lymph nodes was significantly reduced and the disease was under control, and the duration was long. Throughout the treatment, patients were well tolerated. The patient reported in this case had low expression of HER2, had used PD-1 inhibitor and chemotherapy in the past, and was defined as refractory. However, the two-drug combination regimen still showed good efficacy, suggesting that this combination therapy may improve the efficacy of patients with metastatic PUC, despite of low expression of HER2, and may reverse the drug resistance of PD-1 inhibitors and chemotherapy to a certain extent. Further studies are warranted to confirm the effectiveness of Disitamab Vedotin combination with PD-1 inhibitor for patients with metastatic PUC.

## Data availability statement

The original contributions presented in the study are included in the article/supplementary material. Further inquiries can be directed to the corresponding authors.

## Ethics statement

Written informed consent was obtained from the individuals for the publication of any potentially identifiable images or data included in this article. Written informed consent was obtained from the participant/patient(s) for the publication of this case report.

## Author contributions

YZ: Conceptualization, Data curation, Funding acquisition, Writing – original draft. Y-YX: Funding acquisition, Investigation, Writing – original draft. Y-QZ: Conceptualization, Data curation, Funding acquisition, Investigation, Writing – original draft. YC: Supervision, Validation, Writing – review & editing. Z-PL: Supervision, Writing – review & editing.

## References

[1] Janisch F Abufaraj M Fajkovic H Kimura S Iwata T Nyirady P. Current disease management of primary urethral carcinoma. Eur Urol Focus (2019) 5(5):722–34. doi: 10.1016/j.euf.2019.07.001 31307949

[2] Dalbagni G Zhang ZF Lacombe L Herr HW. Female urethral carcinoma: an analysis of treatment outcome and a plea for a standardized management strategy. Br J Urol (1998) 82(6):835–41. 10.1046/j.1464-410x.1998.00878.x 9883221

[3] Swartz MA Porter MP Lin DW Weiss NS. Incidence of primary urethral carcinoma in the United States. Urology (2006) 68(6):1164–8. 10.1016/j.urology.2006.08.1057 17141838

[4] Gakis G Bruins HM Cathomas R Compérat EM Cowan NC van der Heijden AG. European association of urology guidelines on primary urethral carcinoma-2020 update. Eur Urol Oncol (2020) 3(4):424–32. doi: 10.1016/j.euo.2020.06.003 32605889

[5] Dimarco DS Dimarco CS Zincke H Webb MJ Bass SE Slezak JM. Surgical treatment for local control of female urethral carcinoma. Urol Oncol (2004) 22(5):404–9. 10.1016/S1078-1439(03)00174-1 15464921

[6] Lagarde-Lenon MS Aron M. Reprint of: Female Urethral Carcinoma: A contemporary review of the clinicopathologic features, with emphasis on the histo-anatomic landmarks and potential staging issues. Hum Pathol (2023) 133:126–35. doi: 10.1016/j.humpath.2023.02.011 36894368

[7] Visser O Adolfsson J Rossi S Verne J Gatta G Maffezzini M. Incidence and survival of rare urogenital cancers in Europe. Eur J Cancer (Oxford England: 1990) (2012) 48(4):456–64. doi: 10.1016/j.ejca.2011.10.031 22119351

[8] Sui W RoyChoudhury A Wenske S Decastro GJ McKiernan JM Anderson CB. Outcomes and prognostic factors of primary urethral cancer. Urology (2017) 100:180–6. doi: 10.1016/j.urology.2016.09.042 27720774

[9] Gakis G Morgan TM Efstathiou JA Keegan KA Mischinger J Todenhoefer T. Prognostic factors and outcomes in primary urethral cancer: results from the international collaboration on primary urethral carcinoma. World J Urol (2016) 34(1). doi: 10.1007/s00345-015-1583-7 PMC10176500 25981402

[10] Peyton CC Azizi M Chipollini J Ercole C Fishman M Gilbert SM. Survival outcomes associated with female primary urethral carcinoma: review of a single institutional experience. Clin Genitourin Cancer (2018) 16(5):e1003–13. doi: 10.1016/j.clgc.2018.05.012 29859736

[11] Lee W Yu J Lee J-L Kim YS Hong B. Clinical features and oncological outcomes of primary female urethral cancer. J Surg Oncol (2022) 125(5):907–15. doi: 10.1002/jso.26790 35050502

[12] Sheng X Yan X Wang L Shi Y Yao X Luo H. Open-label, multicenter, phase II study of RC48-ADC, a HER2-targeting antibody-drug conjugate, in patients with locally advanced or metastatic urothelial carcinoma. Clin Cancer Res (2021) 27(1):43–51. doi: 10.1158/1078-0432.CCR-20-2488 33109737

[13] Yang S Wan S Zheng D Yin Y Li W Shang P. Treatment and outcomes of primary urethral cancer. Asian J Surg (2022) 45(9):1726–7. doi: 10.1016/j.asjsur.2022.01.014 35165024

[14] Son CH Liauw SL Hasan Y Solanki AA. Optimizing the role of surgery and radiation therapy in urethral cancer based on histology and disease extent. Int J Radiat Oncol Biol Phys (2018) 102(2):304–13. doi: 10.1016/j.ijrobp.2018.06.007 29908944

[15] Thomas A Teicher BA Hassan R. Antibody-drug conjugates for cancer therapy. Lancet Oncol (2016) 17(6):e254–62. doi: 10.1016/S1470-2045(16)30030-4 PMC6601617 27299281

[16] Shi F Liu Y Zhou X Shen P Xue R Zhang M. Disitamab vedotin: a novel antibody-drug conjugates for cancer therapy. Drug Deliv (2022) 29(1):1335–44. doi: 10.1080/10717544.2022.2069883 PMC9090390 35506447

[17] Li L Xu MZ Wang L Jiang J Dong LH Chen F. Conjugating MMAE to a novel anti-HER2 antibody for selective targeted delivery. Eur Rev Med Pharmacol Sci (2020) 24(24):12929–37. doi: 10.26355/eurrev_202012_24196 33378043

[18] Xu Y Wang Y Gong J Zhang X Peng Z Sheng X. Phase I study of the recombinant humanized anti-HER2 monoclonal antibody-MMAE conjugate RC48-ADC in patients with HER2-positive advanced solid tumors. Gastric Cancer (2021) 24(4):913–25. doi: 10.1007/s10120-021-01168-7 PMC8205919 33945049

[19] Huang L Wang R Xie K Zhang J Tao F Pi C. A HER2 target antibody drug conjugate combined with anti-PD-(L)1 treatment eliminates hHER2+ tumors in hPD-1 transgenic mouse model and contributes immune memory formation. Breast Cancer Res Treat (2022) 191(1):51–61. doi: 10.1007/s10549-021-06384-4 34657203

[20] Zhou L Xu H Li S Yan X Li J Wu X. Study RC48-C014: Preliminary results of RC48-ADC combined with toripalimab in patients with locally advanced or metastatic urothelial carcinoma. (2022) 40(6_suppl):515–5. doi: 10.1200/JCO.2022.40.6_suppl.515

[21] Chen M Yao K Cao M Liu H Xue C Qin T. HER2-targeting antibody-drug conjugate RC48 alone or in combination with immunotherapy for locally advanced or metastatic urothelial carcinoma: a multicenter, real-world study. Cancer Immunol Immunother (2023). doi: 10.1007/s00262-023-03419-1 PMC10264489 36897337

